# In-Memory-Computing Realization with a Photodiode/Memristor Based Vision Sensor

**DOI:** 10.3390/ma14185223

**Published:** 2021-09-10

**Authors:** Nikolaos Vasileiadis, Vasileios Ntinas, Georgios Ch. Sirakoulis, Panagiotis Dimitrakis

**Affiliations:** 1 Institute of Nanoscience and Nanotechnology, National Center for Scientific Research “Demokritos”, 15341 Agia Paraskevi, Greece; 2 Department of Electrical and Computer Engineering, Democritus University of Thrace (DUTh), 67100 Xanthi, Greece; vntinas@ee.duth.gr (V.N.); gsirak@ee.duth.gr (G.C.S.)

**Keywords:** resistive random-access memory (RRAM), resistance switching, silicon nitride, memristor, vision sensor, photodiode, crossbar, in-memory computing, edge computing, dot product engine, IoT, SPICE

## Abstract

State-of-the-art IoT technologies request novel design solutions in edge computing, resulting in even more portable and energy-efficient hardware for in-the-field processing tasks. Vision sensors, processors, and hardware accelerators are among the most demanding IoT applications. Resistance switching (RS) two-terminal devices are suitable for resistive RAMs (RRAM), a promising technology to realize storage class memories. Furthermore, due to their memristive nature, RRAMs are appropriate candidates for in-memory computing architectures. Recently, we demonstrated a CMOS compatible silicon nitride (SiN_x_) MIS RS device with memristive properties. In this paper, a report on a new photodiode-based vision sensor architecture with in-memory computing capability, relying on memristive device, is disclosed. In this context, the resistance switching dynamics of our memristive device were measured and a data-fitted behavioral model was extracted. SPICE simulations were made highlighting the in-memory computing capabilities of the proposed photodiode-one memristor pixel vision sensor. Finally, an integration and manufacturing perspective was discussed.

## 1. Introduction

During the last decade, it became apparent that created data are increasing rapidly, requesting revolutionary solutions when memory and storage is concerned. These needs are more demanding in case of Internet of Things (IoT) applications and the corresponding IoT sensors that produce zettabytes of data nowadays. The most straightforward approach to tackle the uprising urgent issue is the local pre-processing of the unstructured data generated by the IoT sensors in an edge-based sense [1,2,3,4]. Such a promising solution will eventually minimize the requesting power consumption of the corresponding IoT applications and at the same time advance the overall computing in terms of energy efficiency. However, following conventional digital design approaches involving either specialized signal processors or more generic microcontrollers does not prove as promising as expected and especially when power consumption is highly demanded [5]. The next obvious step of utilizing a more specific-oriented CMOS-based design can be significantly enhanced with the addition of novel nanoelectronic devices with memory abilities, namely memristors, to be combined with the ΙοΤ sensors. To further investigate the promising aspects of such a hardware approach enabling also in-memory computation at IoT sensors, special interest is on vision sensors as a fine candidate for edge computing. The vision sensors, when integrated with such processing hardware, are enabled to provide low-power computing abilities for a number of various applications instead of only capturing the picture [6]. Nevertheless, from this perspective, we propose a photodiode-memristor (1D1M) vision sensor that integrates, in series, the photodiode with a memristor in a crossbar (Xbar) array. This rather simple architecture gives the potential of analog non-volatile image storage and massive parallel read operations with simultaneous dot product computations, reducing the need for an expensive processor unit for basic image-processing tasks. The proposed 1D1M sensor array could be considered as a light-to-resistance converter, image storage, as well as dot product accelerator. This method is more attractive for implementation compared to a phototransistor because when implementing the memristive element with a photodiode, the fabrication complexity is reduced. Additionally, the parasitic currents through base-emitter junction disappear during memristance measurement operations.

The integration of memristors in photodiode circuits was demonstrated in [7], where the memristor was used as a photocurrent integrator in the readout integrated circuit. Furthermore, in [8] a vision sensor with adaptive background subtraction as the basic engine for object tracking was implemented. There, memristors were used to store the dynamic boundaries, outside which the behaviour of the photo-generated signal is recognized to be anomalous. Moreover, hybrid RRAM-CMOS vision sensors have also been proposed in literature integrating commercial photodiode pixel architectures combinations with memristors [9], while in these architectures, each pixel requires three MOSFETs [10].

In this disclosure, a CMOS-compatible SiN memristor [11,12] is used because of the SiN-based insulators immunity against environmental oxygen-related reliability effects, metal ion diffusion, and humidity. In addition, silicon nitride nonvolatile memories (NVM) have been well-established and various charge-trapping memory devices are commercial nowadays (e.g., BiCS, SONOS). Their acceptance by the community is mainly attributed to the intrinsic bulk defects that act as trapping levels for both electrons and holes [13,14,15]. The resistive switching and conduction mechanism in SiN memristors is directly related to these intrinsic defects, as has been shown by several research groups [16,17,18]. Recently, we demonstrated the role of the SiN-traps to form the various resistance levels [19] and to use them in practical applications like true random number generators [20]. Moreover, the scalability [21] and the neuromorphic aspects [22] of these memristors have also been demonstrated and are very attractive compared to other RRAM technologies, which are considered as one of the most promising candidates for emerging nonvolatile memories. More specifically, RRAMs, thanks to their premium characteristics in terms of scalability, simplicity, and low energy storage, are implemented successfully in Xbar architectures aiming at the smallest memory cell, 4F^2^, where F is considered as the minimum feature size that is obtained by lithography [23], and furthermore, due to their multiply and accumulation current characteristics, various RRAM Xbar arrays implementations of in-memory computing [24,25] and neuromorphic computing [26] have been already proposed in literature. In terms of unconventional computing, Resistive Switching (RS) devices have been demonstrated as adequate memristors able to store qubits in quantum simulators [27]. As a result, the proposed Xbar design and integrations of memristors with photodiodes for image sensing and in-memory processing, alike edge computing, sounds promising, and the presented SPICE-based simulation results reveal its successful implementation. More specifically, 28 × 28 1D1M Xbar circuit array SPICE simulation results exploit the in-memory processing abilities of the proposed vision sensor.

## 2. Silicon Nitride Memristor Devices as Analog Switches

### 2.1. Device Fabrication 

**Figure 1:**
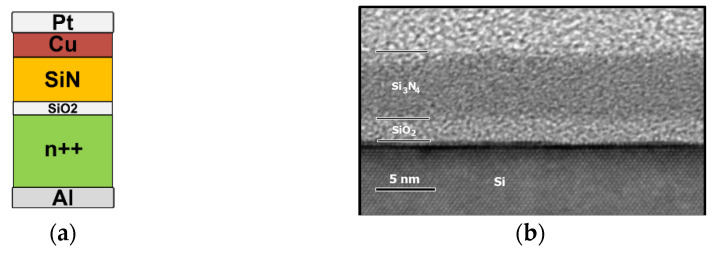
(**a**) Schematic representation of the fabricated and examined memristive device structure. (**b**) XTEM micro-image of the fabricated SiN_x_ and SiO_2_ layers on n^++^-Si wafer.

On n^++^-Si wafer, where ρ < 0.003 Ωcm, a 2 nm SiO_2_ layer was thermally grown. Subsequently, a 6 nm SiN_x_ layer was deposited by LPCVD at 810 °C, using ammonia (NH_3_) and dichlorosilane (SiCl_2_H_2_) gas precursors. The SiO_2_ layer was selected to be placed in between SiN_x_ and Si for two main reasons. In the first place, to provide us with the ability to further control carriers’ injection from n^++^ -Si bottom electrode (BE) to SiN_x_. The second obvious reason is to enable us to succeed the retention increment of the resistance levels with the addition of a higher energy barrier; subsequently, the trapped carriers’ leakage from SiN_x_ to Si-BE was accordingly mitigated. Moreover, the interface of the SiN_x_/Si presents significantly lower quality concerning the interfacial defects when compared with the interface between the SiO_2_ (thermal dry oxide) and the Si-BE. As a result, it is expected that owing to the subsequent thermally activated exchange, in other words trapping/de-trapping of the carriers between Si-BE and SiN_x_, as well as the aforementioned interfacial traps lower concentration, the intermediate SiO_2_ layer will be able to efficiently suppress the electronic noise. Si doping is high enough to minimize the substrate depletion/ inversion capacitance, which is attributed to transient parasitic effects under pulse operation conditions. Nevertheless, the reduction of Si doping leads to self-rectification [28,29] in the I-V characteristics, which is very attractive for memory operation. Furthermore, with the help of photolithography and metal lift-off, we were able to define the Top-electrode (TE) as well. In more details, the TE metallization process corresponds to a sputtered 30 nm Cu layer also covered by 30 nm Pt in order to avoid oxidation of Cu [12]. A schematic representation of our device is presented together with an XTEM micro-image, which is shown in Figure 1. The later allows for the measurement of the SiN_x_ and oxide layer thickness, which are (6.2 ± 0.3) nm and (2.1 ± 0.3) nm, respectively. Clearly, the nitride layer is amorphous, homogeneous, and without meaningful surface and interface roughness.

### 2.2. Analog Resistive Switching Characteristics 

**Figure 2:**
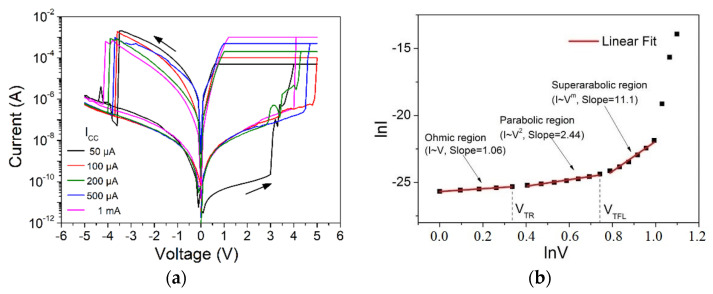
(**a**) Typical bipolar switching behavior for SiN_x_ Memristor. Different resistance levels achieved under different current compliance values. (**b**) Analysis of typical I-V sweep characteristic for SiN_x_ memristor during SET following the SCL conduction mechanism.

The origin of the resistance switching (RS), as shown in Figure 2a, is not still well established. Except [30] where the most probable mechanism is attributed to the movement of protons due to the large concentration of hydrogen atoms, all research results converge to the conclusion that RS originates from a trap-assisted mechanism [16,18]. This is mainly due to the different deposition techniques that affect the thermodynamic parameters of the defect formation. In our case, as clearly shown in Figure 2b, the space charge limited conduction (SCLC) mechanism was best fitted to our I–V measurements, and this is the most common mechanism found in literature for SiN_x_ memristors [18]. Initially, the tunneling of electrons to short-range defects in the nitride layer (Ohm’s law, I~V) is apparent, and as the voltage increases to more than the threshold value V_TH_, traps deeper in the layer are filled, causing the transition from linear to parabolic I–V dependence [12]. When all traps are filled (trap-filled region, TFL), at V_TFL_, the current suddenly increases (I~V^n^, n > 2). The calculated slopes in the double logarithmic I–V plots correspond to the values of the voltage exponent, which for the linear and parabolic regions, range from 0.99–1.06 and 2.01–2.57, respectively. This observed variability can be attributed to the presence of the randomly distributed traps inside the SiN_x_ material and the interfaces, and fully agrees with previous published results in SiN MIM [18]. According to the SCLC theory [12], the concentration of traps can be estimated from

(1) $$N_{t}=\frac{2\varepsilon V_{TFL}}{qd^{2}}$$

where *ε* is insulator’s vacuum dielectric constant, *q* is the fundamental electronic charge, while *d* and *V_TFL_* denote the insulator’s thickness and the trap-filled limit voltage, respectively. According to (1), the trap concentration in SiN_x_ layers was estimated 1.2 × 10^20^ cm^−2^, which is a typical value for such LPCVD silicon nitride material. According to our recent work [19], the energy levels of the predominant traps in a typical 200 kΩ resistance level were found at ca. 0.6 eV to ca. 0.7 eV below the conduction band of silicon nitride, which is in accordance with [13,15]. These trapping levels correspond to adjacent nitrogen traps in the nitride bandgap. These traps originated either due to the breakage of = N – H and ≡ Si – N = bonds [13] or due to hydrogen incorporation in silicon-silicon dangling bonds [15]. In Figure 2a, the I–V characteristics obtained by DC sweep for the investigated SiNx memristors are presented. Obviously, different resistance levels could be achieved under different current compliance values, in which it is evident that SiNx memristor is not a bistable memory element but has analog switching characteristics.

**Figure 3:**
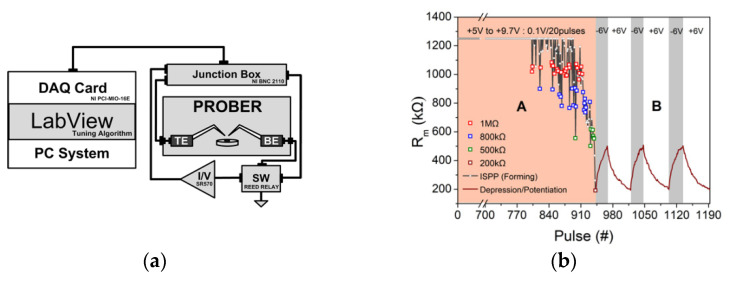
(**a**) Block diagram of the switching measurement experimental setup, (**b**) ISSP forming procedure is illustrated in region A, and three potentiation/depression cycles under pulses of 1 μs width and ±6 V amplitude are illustrated in region B.

In order to achieve accurate investigation of memristor’s dynamics, we assembled a DAQ-card, namely a NI-PCIO-MIO-16E card that was attached to an I/V converter, namelySR570 through a low-noise junction box, i.e., NI BNC 2110, all presented in the block diagram of Figure 3a. In addition, a wafer probe station was utilized to set the aforementioned memristor device with the help of triaxial cables for the application of required voltage pulses and measurement of the corresponding output currents. A LabView environment was finally applied to control the presented measurement experimental setup, enabling any tuning pulsing sequence of arbitrary waveform as well the switching between I/V and ground, or in other words Read and SET/RESET, respectively. The later operation was realized by using a reed relay, namely the HE3321X050 reed relay. It should be noticed that all the following measurements were conducted without the appliance of any current compliance mechanism. 

For our experiments, we examined memristors with area 100 μm × 100 μm. ISPP, incremental step pulse programming technique, was utilized to achieve proper switching from High Resistance State (HRS) to 200 KΩ [31]. This was achieved with pulses of 1 μs duration and amplitude located in the range of +5 V to +9.7 V, grading with 0.1 V steps for every 20 pulses. The repetition rate/frequency of the applied pulses was 33 Hz, while the resistance was measured after each ISSP produce pulse by pulsed current (0.1 V/200 μs). The forming procedure is shown in Figure 3b (region A). Obviously, four significant resistive states (0.2 MΩ, 0.5 MΩ, 0.8 M, and 1 MΩ) were revealed in the examined devices and are marked in Figure 3b with colored squares symbols. According to the literature [19,32], the observed resistance modification results from the redistribution of the silicon nitride traps inside the material, performed in a progressive manner, forming a conductive filamentary region, enabling the ejection (or injection) of charge carriers into these traps, and modulating the resistance of filament [19]. Voltage pulses with different heights are attributed to exchange carriers with traps of different activation energy; the larger the pulse height, the larger the trap energy probed. In this framework, the four stable resistance states mentioned previously can be interpreted. Recently, Yonai et al. [33], using similar devices, proved that pulsing frequency and duty cycle during potentiation drastically affect the forming and endurance characteristics.

Following the ISPP forming procedure, consecutive pulse trains (1 pulse train = 20 pulses) of width 1 μs and amplitude ±6 V, result in fine modulation of resistance from 0.2 MΩ to 0.5 MΩ (depression) and back (potentiation). Experimental results of potentiation and depression cycles are presented in Figure 3b (please check region B), indicating that the traps redistribution in the filamentary area of the nitride layer can be accurately controlled. Furthermore, it is demonstrated that the examined memristors can mimic the operation of neuronal synapses.

### 2.3. Analog Resistive Switching Behavioral Modeling 

**Figure 4:**
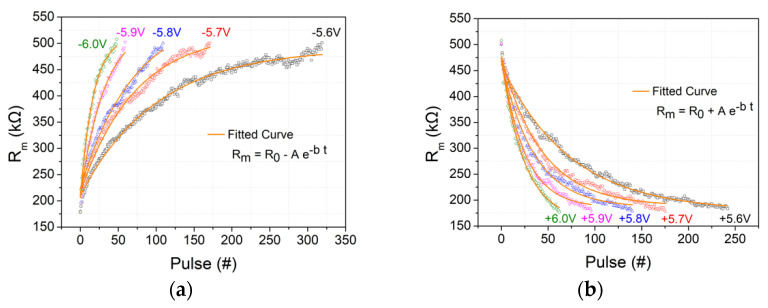
Single exponential experimental fitting on (**a**) depression and (**b**) potentiation measured data for ±5.6 V to ±5.9 V/1 μs pulses.

In order to simulate any circuit employing the aforementioned fabricated SiN-based memristor, an accurate model for the resistance switching dynamics is required. For this purpose, single memristor resistance measurements were performed by utilizing sets of 20 pulses, pulse trains, with alternating polarities. The results of this procedure are depicted in Figure 4. It should be noted that the aforementioned pulse trains have different amplitudes and modulate the resistance of the tested devices 0.2–0.5 MΩ, accordingly. Clearly, the exponential relation (2) can sufficiently fit on the depression/potentiation measured data.

(2) $$R_{m}\left(t\right)={\bar{R}}_{0}+\bar{A}e^{-bt}$$

**Figure 5:**
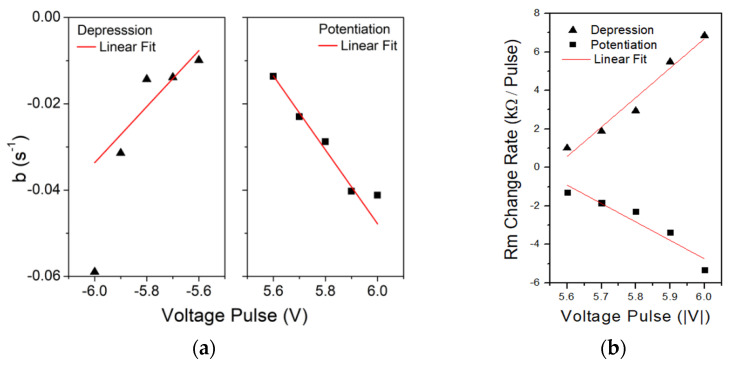
(**a**) Linear dependence of the exponent *b*. The values of *b* were extracted from fitting of relation (2) to experimental data as shown in Figure 4. (**b**) Average rate of R_m_ change for different potentiation/depression pulse amplitudes.

**Figure 6:**
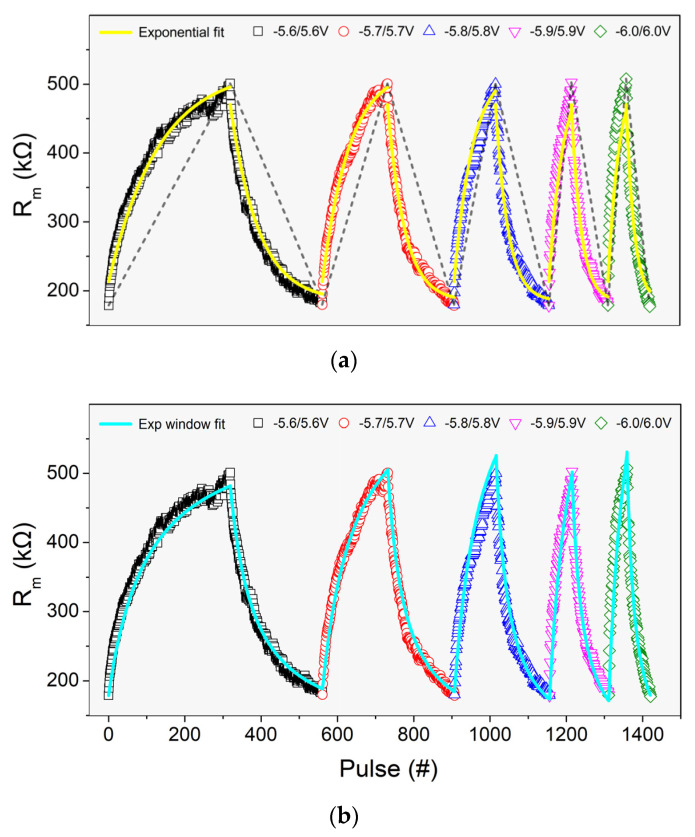
(**a**) Exponential (upper, yellow curve) and (**b**) windowed exponential (lower, cyan curve) fitting models.

Experimental data fitting on (2) are presented with solid lines in Figure 4. The applied pulse has the same polarity with parameter A, while the pulse amplitude (±5.6 V to ±5.9 V) is related in a linear manner with parameter b, as shown in Figure 5. Evidently, *R_m_*(*t*) evolution with respect to applied potential pulse V can be modeled accurately, in these ranges, by Equation (2). Average rate of *R_m_* change per different voltage pulse is also presented in Figure 5 (dashed lines slopes of Figure 6) and gives a better intuitive picture of the potentiation/depression phenomenon. It was observed that for every volt change (absolute value) on potentiation/depression pulse amplitude, the rate of Rm change increases by about 1 kΩ resistance per pulse. 

It is clear that in the case of ±6 V, the fitting *R_m_*(*t*) turns to be less accurate and close to the extremum region of our data. To tackle this issue, a voltage window is selected to be applied to the aforementioned exponential relation that describes *R_m_* evolution.

(3) $$\frac{dR}{dt}=s\left(v\right)\times f\left(R,r\left(v\right)\right)$$

(4) $$s\left(v\right)=\{\begin{array}{c} A_{p}\left(-1+e^{\frac{\left|v\right|}{t_{p}}}\right),\;v>0\\ A_{n}\left(-1+e^{\frac{\left|v\right|}{t_{n}}}\right),\;v\leq 0 \end{array}$$

(5) $$f\left(R,v\right)=\{\begin{array}{c} {\left(r_{p}\left(v\right)-R\right)}^{2},\;v>0\\ {\left(R-r_{n}\left(v\right)\right)}^{2},\;v\leq 0 \end{array}$$

The proposed method with the window applications was successfully utilized before in a related work [34]. More specifically, the model Equation (3) includes a state function  s(v) (4) for the evolution of the state of the memristor multiplied by a mathematical window f(R,v) (5) to limit the state within a certain resistance range. In addition, the window function f(R,v) uses the internal Equation (6) to calculate the target resistance r(v), which varies depending on the width of the applied voltage:

(6) $$r\left(v\right)=\{\begin{array}{c} r_{p}\left(v\right)=a_{0,p}+a_{1,p}\cdot v,\;v>0\\ r_{n}\left(v\right)=a_{0,n}+a_{1,n}\cdot v,\;v\leq 0 \end{array}$$

where, v and R are the applied voltage and the (resistance) state of the memristor, respectively, while all other variables are fitting parameters [34]. 

Table 1 shows the values of the fitting parameters of the exponential windowed model as they emerged though fitting in our data with MATLAB’s global optimization toolbox. A comparative view of the two fitting models is shown in Figure 6, where the yellow line presents the single exponential fitting model, and the cyan line, the windowed exponential model, respectively. For nearly any applied voltage, the fitting accuracy performed for the windowed exponential model is significantly improved. 

## 3. One Photodiode One Resistor (1D1M) Vision Sensor

### 3.1. Architectural Overview

**Figure 7:**
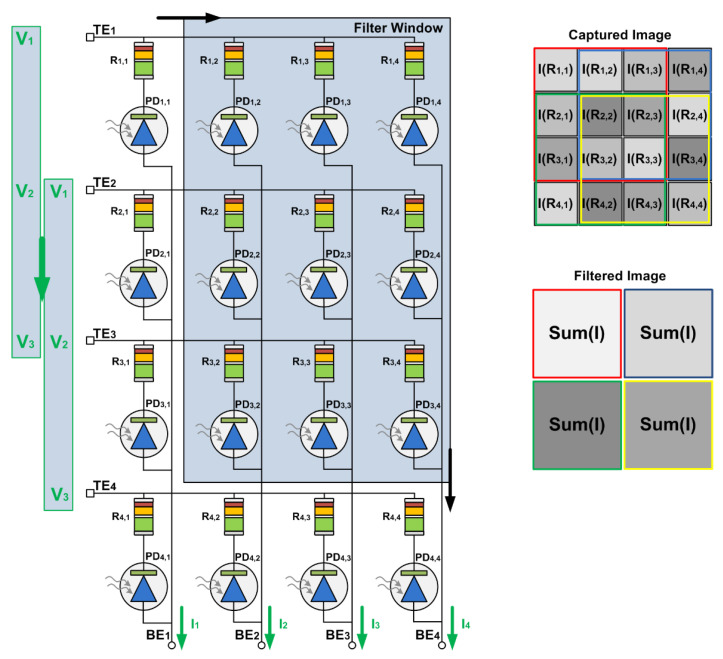
1D1M vision sensor architecture, where the read mask together with the virtual corresponding filter window is depicted with boxes colored with a light blue color. The moving read mask with the corresponding virtual filter window is marked by light blue boxes. Each pixel of the filtered image is color marked with the same color as the corresponding convolutional region of the captured image.

Herein, a 1D1M architecture is disclosed, comprising the implementation in series of a photodiode with the proposed SiN memristor integrated in a Xbar array with common TE per row and common BE, namely the photodiode’s anode per column, respectively. In Figure 7, a detailed symbolic representation of the disclosed architecture is presented.

In the following, the 1D1M vision sensor operation is presented. As already presented in the corresponding region A of Figure 3b after a forming procedure (see Figure 3b—Region A), which will take place either to initialize the sensor or if the formed filament breaks, the first operation of the vision system is to erase (or reset, ERS) the memristors’ resistance to the initial state of 0.5 MΩ, which can be succeeded with negative pulses on TEs nodes while BEs are grounded. In correspondence, the second step is programming (SET or PGM), with the PD array light exposure. As a result, when various light intensities are going to be applied on PDs, this will result in various voltage drops at the memristors’ electrodes for the same V_TE_, thus causing correspondingly various resistance changes. In other words, memristance in the range of 0.2–0.5 MΩ is considered by the light intensity conversion and, in such a manner, an image can be easily stored in the Xbar array. It is clear enough that the light sensitivity of the vision sensor can be controlled with the application of various V_TE_ voltages. Finally, the read image (READ) operation can be succeeded by applying row-by-row V_READ_ = −(V_bi_ + 0.1)V on TEs and measuring the corresponding currents on BEs. The produced sensory image will be in the range of [V_READ_/R_m,MAX_, V_READ_/R_m,MIN_]. The READ operation can be completed in N steps equal to the number of rows.

In-memory computing capabilities can also be utilized through this architecture. In the previous final step of the READ operation, $V_{READ}$ voltages can be applied on multiple rows at the same time, which will lead to accumulating currents flow on BEs. Figure 7 presents this functionality. Multiple $V_{READ}$ voltages are applied as a moving mask $\left[V_{1}\;V_{2}\;V_{3}\right]$ on the TEs, while BEs currents export the accumulated dot products $I_{j}=\underset{column}{\sum }V_{READ}\left(i,j\right)*[1/R_{m}\left(i,j\right)]$ of the activated rows (green arrows). With external summation of these currents in groups of mask-size (in this example by 3), a filtered image can be produced. This implementation gives a fundamental pre-process functionality to the vision sensor and increases its portability, excluding the need for a more complex processor for in-the-field applications. The drawback of the method is when the filter mask is not uniform and each different mask column should be reapplied to the sensor. In this case, the processing time will be increased linearly by a factor of n equal to number of mask columns needed to be applied to the sensor. Finally, masks with ambipolar values are not allowed because only one current direction is allowed through the photo-diode during the READ operation.

### 3.2. Integration Perspectives

**Figure 8:**
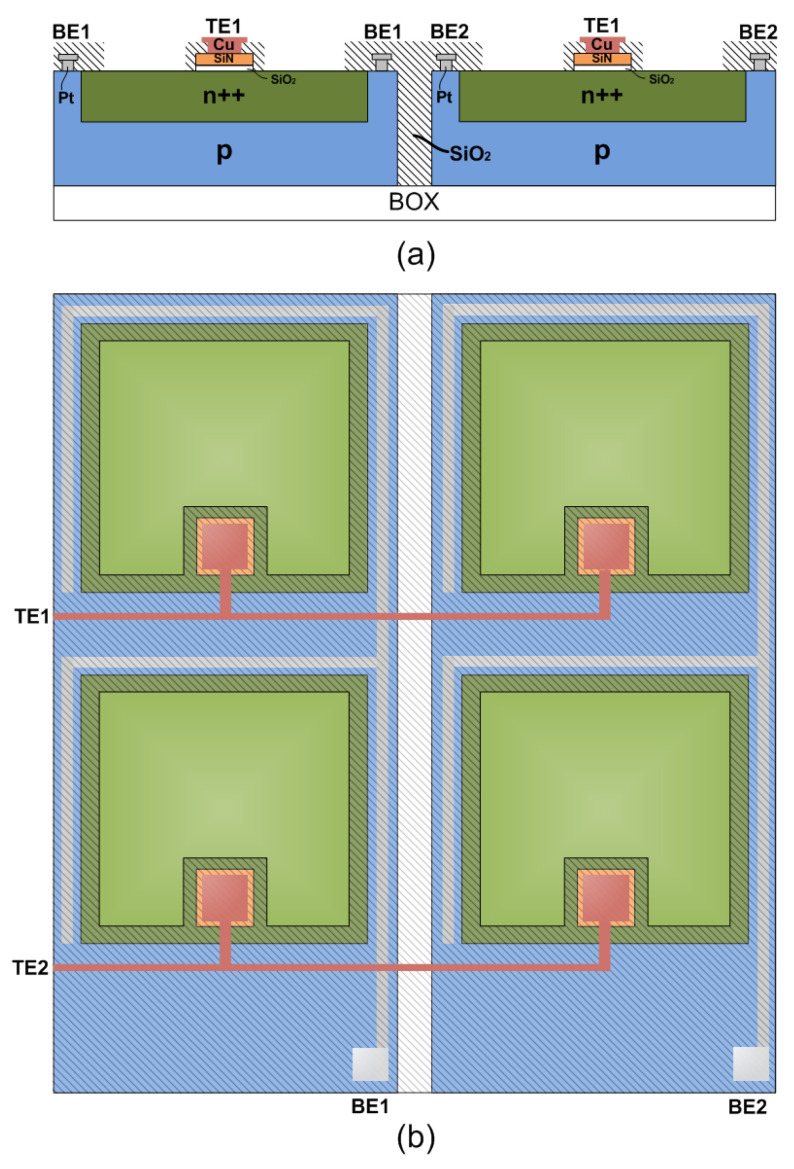
1D1M vision sensor’s schematic layout: (**a**) 2 adjacent pixels of the same row cross section view; (**b**) 2 × 2 sensor’s pixel array top view. 3.3. SPICE Simulated in-memory-computing operations.

As we mentioned previously, the SiN memristor is a fully compatible CMOS process device and for this reason the SiN-PD array (see Figure 8) integration in a manufacturing environment is feasible. To avoid pixel crosstalk as well as the formation of parasitic bipolar transistor between adjacent pixel, silicon-on-insulator (SOI) substrates are more preferable. Figure 8a illustrates the cross section of two adjacent pixels in the same row, while Figure 8b presents the schematic layout of a 2 × 2-unit cell of 1D1M presented vision sensor. The required processing steps for this integration are described as follows. The fabrication starts with the formation of n^++^-Si region by ion implantation, followed by the deposition of the dielectric stack (SiO_2_ and SiN_x_) on p-type SOI wafer. Next, pixel dielectric isolation takes place through silicon dry etching till the buried oxide (BOX) and the uncovered area is filled by TEOS deposition. Following, BE contact metallization (Al) through lithography and metal lift-off take place. Then, the Al electrodes are covered by low-temperature oxide (LTO). After pixel active area definition by lithography, LTO is stripped from the PD n^++^ region. Finally, the TE formation (Cu/Pt) is performed through lithography and metal lift-off.

Speed and responsivity optimization will be obtained through specialized PD design in the future. The PD’s p-n junction capacitance will be adjusted, which also regulates the bandwidth. The most important parameter affecting the sensor’s efficient operation is the dark current of the PD, and specific optimization steps are required. Another critical parameter is the coupling capacitance between BE and TE metal lines, which requires a precise calculation of the geometrical characteristics and precise routing. All these optimizations will be achieved through comprehensive TCAD modeling as part of our ongoing research work in these topics.

### 3.3. SPICE Simulated in-Memory-Computing Operations

**Figure 9:**
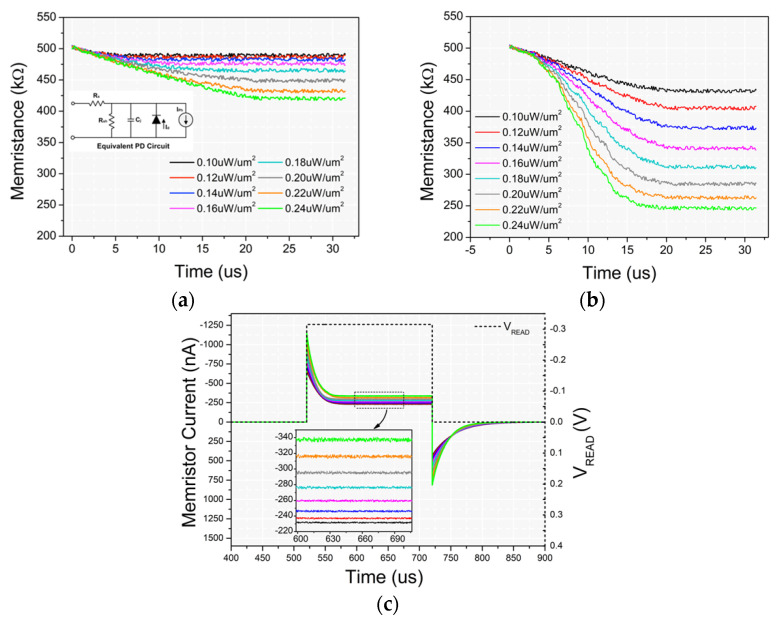
Memristance change as calculated on SPICE simulation for eight PGM operations with eight different light intensities on a single 1D1M element with (**a**) constant *V_TE_* bias and (**b**) V_TE_ increment by 0.4 V/three light pulses. (**c**) Memristor currents for eight READ operations of 500 μs after the previous eight PGM operations. The inset in (**a**) displays the equivalent PD subcircuit was used in SPICE simulation.

SPICE simulations are made to evaluate the functionality of the proposed sensor’s circuitry. Firstly, the single 1D1M element is simulated. The inset of Figure 9a shows the equivalent PD circuit used for this purpose while the related quantities are described by the following set of equations:

(7) $$R_{S}=\frac{\left(W_{S}-W_{d}\right)\rho }{A}+R_{C}$$

(8) $$C_{J}=\frac{\varepsilon_{si}\varepsilon_{0}A}{W_{d}},\;W_{d}=\sqrt{2\varepsilon_{si}\varepsilon_{0}\mu \rho \left(V_{TE}+V_{bi}\right)}$$

(9) $$I_{Ph}=W_{opt}\times Resp\times A$$

More specifically, series resistance of the photodiode is modeled by (7), where $W_{S}\;(=$300 um) is the thickness of the substrate, $W_{d}\;\left(=0.5\;\mu m\right)$ is the width of the depleted region, $A\;\left(=100\;{\mu m}^{2}\right)$ is the diffused area of the junction, ρ (=3 mΩcm) is the resistivity of the silicon substrate, and R_C_ is the contact resistance. Junction capacitance $C_{J}$ is modeled by (8), where $\varepsilon_{0}\;\left(=8.854\times 10^{-14}\;F/\mathrm{cm}\right)$ is the permittivity of free space, $\varepsilon_{si}\;\left(=11.9\right)$ is the silicon dielectric constant, $\mu \;\left(=1400\;{\mathrm{cm}}^{2}/V\cdot s\right)$ is the mobility of the electrons at 300 K, $V_{bi}\;\left(=0.65\;V\right)$ is the built-in voltage of silicon, and $V_{TE}$ is the applied bias. Photocurrent is given by (9), where $W_{opt}$ is the incident light power and Resp (=0.5) is the responsivity of the PD. Shunt resistance $R_{sh}\;\left(=100\;M\Omega \right)$ is the slope of the current-voltage curve of the photodiode at low voltages, and it is used to determine the noise current in the photodiode with no bias. Finally, for the internal diode of the PD subcircuit, a IN4148 SPICE model is used [35].

Simulation results of a PGM operation on a 1D1M unit are presented in Figure 9a where memresistance changes under 30/1 μs consecutive light pulses on PD’s cathode. A clear separation of eight memristance levels can be achieved by a set of eight different light intensity pulses with power in the range of $W_{opt}\;$ = [0.1 μW/um^2^, 0.24 μW/um^2^]. Additional memristance variability of 5.8 kΩ (the maximum resistance fluctuation in the targeted range of 200–500 kΩ as measured in [19]) was added to the simulation to validate that the states are clearly separated. It is worth to mention that, an important tweak for better resistive state separability, as it is revealed through the simulation process, was the increase of the $V_{TE}$ bias after each light pulse, due to significant voltage drop on the memristor device. More specifically, as shown in Figure 9b, $V_{TE}$ was increased by 0.4 V after every three light pulses and now the three last memristance levels popped out while the overall separation was obviously better. With this method, the memristor bias is kept above its switching voltage threshold for a larger period and its memristance change keeps on. The required increase of *V_TE_* can be easily implemented with a DAC converter. Figure 9c shows the transient responses of eight different READ operations on a 1D1M element after the previous PGM operations with the eight different light power conditions.

**Figure 10:**
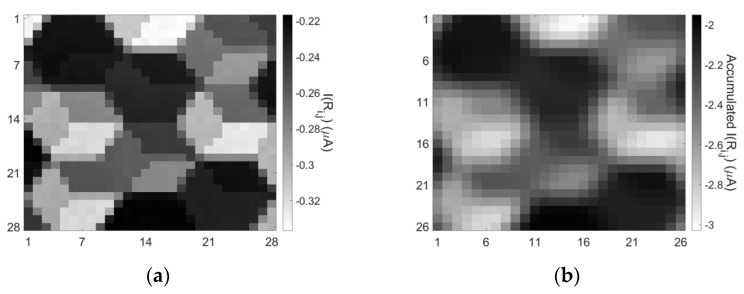
28 × 28 1D1M vision sensor’s in-memory-computing SPICE simulation results for (**a**) an original image as was captured by the sensor and read row-by-row after 30 discrete light pulses on PDs. (**b**) A READ operation simultaneously with mean filtering.

Proposed sensor’s in-memory computing properties were demonstrated through SPICE simulation on a 28 × 28 1D1M crossbar circuit array. In Figure 10a,b, in-memory-computing SPICE simulation results for an image capture and READ operation and a READ operation simultaneously with mean filtering are presented, respectively. In all experiments, the memristors of the 28 × 28 array were initialized at the high resistive state (500 kΩ) and then light pulses were emitted on PDs with eight discrete power levels as shown previously. For the first READ operation of the captured abstract image, a V_READ_ = –(0.1 V + V_d_) was applied row-by-row and the currents measured on BEs. We did not simply add V_bi_ to V_READ_ because voltage drop V_d_ in IN4148 diode model is not equal to V_bi_ under forward bias in-series with resistance. Thus, V_d_ (=0.215 V) was calculated from the operating point of the diode for the memristive state of 350 kΩ and added to V_READ_. With this method, an equal distribution of reading potential around 0.1 V for every memristive state can be achieved while avoiding memristor’s non-linearities. For the in-memory-computing filtering operation, a size-3 mask [V_READ_, V_READ_, V_READ_]^T^ was used and shifted on arrays TEs. For this case, the corresponding accumulated currents were collected from the BEs and summed externally in groups of three. In both cases, eight-level images were exported with clearly separable current levels. Finally, Table 2 shows a comparison between fundamental characteristics similar to our work in memristive in-sensor computing architectures proposed in the latest literature.

## 4. Conclusions

In this work, the potentiation/depression characteristics of a SiN_x_ memristor were measured and modeled, successfully mimicking the neuronal synapses. The architecture of one photodiode–one memristor was presented and simulated. A 1D1M crossbar sensor array was developed and its in-memory computing properties like filtering were demonstrated through SPICE simulations. Finally, an integration and manufacturing perspective was discussed. 

## Figures and Tables

**Table 1:** Fitting values for the exponential windowed model.

Potentiation–Positive Pulses		Depression–Negative Pulses	
$A_{p}\;\left(\Omega \;s^{-1}\right)$	$-8.852\times 10^{-8}$	$A_{n}\;\left(\Omega \;s^{-1}\right)$	0.9085
$t_{p}\left(V\right)$	0.4277	$t_{n}\left(V\right)$	214.06
$a_{0,p}\;\left(\Omega \right)$	$748.5\times 10^{3}$	$a_{0,n}\;\left(\Omega \right)$	$-4.088\times 10^{6}$
$a_{1,p}\;\left(\Omega \;V^{-1}\right)$	$-115.4\times 10^{3}$	$a_{1,n}\;\left(\Omega \;V^{-1}\right)$	$-833.6\times 10^{3}$

**Table 2:** In-sensor computing concepts based on RRAM devices.

Technology [Reference]	NVH RRAM-CMOS Architecture [9]	Networking Retinomorphic Sensor [36]	UMV 2D Material Image Sensor [6]	ATO Machine Vision Processor [37]	AFV Memory System [38]	1P1R Image Sensor [39]	This Work
**Biological** **System** **emulation**	Retinal bioarchitecture	Human Retina	No	Human vision system	Human visual memory	No	No
**CMOS** **process** **compatibility**	Only Pixel Array	Not compatible	Not compatible	Not compatible	Not compatible	All structure	All structure
**Memristive structure**	Hexagonal circuitry	1T1R crossbar	1PD-1FGT crossbar	MoS_2_ photo-FET crossbar	1SMW-1R array	1T1R crossbar	1D1R crossbar
**Memristive** **element**	Pt-Hf-Ti VTEAM Model	Pt/Ta/HfO_2_/Ta	hBN-Au-Al_2_O_3_ (Floating gate memory)	FET PCC ^(1)^	Ni-Al_2_O_3_-Au	SiN_x_ Experimental data model	SiN_x_ Experimental data model
**Analog** **Resistive states**	2 levels On/Off	[0 mA, 4 mA]: [0 V, 0.4 V] discrete levels not mentioned	discrete levels not mentioned	4 discrete levels	2 levels On/Off	16 discrete levels	8 discrete levels
**Photosensitive element**	PN photodiode	WSe_2_/h-BN/Al_2_O_3_ phototransistor	WSe_2_ photodiode	MoS_2_ photo-FET	In_2_O_3_ SMW ^(4)^	NPN-BJT phototransistor	PN photodiode
**Development stage**	IC Mask Layout design and sims	Fabricated	Fabricated	Fabricated	Fabricated	Simulation	Simulation
**Estimated power** **consumption**	7.8 μW ^(2)^	N/A	N/A	1.65 μW ^(3)^	N/A	N/A	N/A
**Estimated IC pixel area size**	N/A	N/A	17 × 17 μm^2^	300 × 300 μm^2^	0.5 × 0.5 cm^2^	10 × 10 μm^2^	10 × 10 μm^2^
**Sensor size** **investigated**	128 × 128 PDs 16 × 16 RRAMs arrays	N/A	27× 3 × 3 Pixel (PD + FGT) array	32 × 32 photo-FET array	10 × 10 (SMW + RRAM) array	32 × 32 (PD + RRAM) array	28 × 28 (PD + RRAM) array
**In-memory** **computing** **application Demo**	Retinal Line spread function approximation	Edge enhancement, stylization and recognition	ANN classifier	Edge Detection, Embossing, Blur and Visual recognition	N/A	Mean Filtering And Edge Detection	Mean Filtering

^(1)^ Persistent photoconductivity; ^(2)^ Single Cell; ^(3)^ Average per input image; ^(4)^ SMW = Semiconductor micrometer-sized wires.

## Data Availability

The data presented in this study are available on request from the corresponding authors.
